# Supplementary material for: Utilization of long-acting contraceptive methods and associated factors among female healthcare providers in South Wollo Zone hospitals, Northeast, Ethiopia. A cross-sectional multicenter study
Source: PLOS Glob Public Health. 2023 Mar 27;3(3):e0001692. doi: 10.1371/journal.pgph.0001692 (PMC10042362; doi:10.1371/journal.pgph.0001692)
Supplement: Attachment — Submitted filename: A rebuttal letter of responses.docx [file pgph.0001692.s002.docx]

***A rebuttal letter of responses***:

Journal Requirements:

1. We have made thoroughly copyedit our manuscript for language usage, spelling, and grammar.
2. Please amend your detailed Financial Disclosure statement

*Wollo University supported to conduct this study.  The funders had no role in study design, data collection and analysis, decision to publish, or preparation of the manuscript.*

3. Data availability

*The data cannot be made publicly available for ethical or legal reasons. It will be available at request.*

4. Please ensure that the Title in your manuscript file and the Title provided in your online submission form are the same.

*Corrected*

5. Please provide separate figure files in .tif or .eps format and removed from the manuscript file.

*Corrected*

**Reviewers' comments’ response**

**Reviewer 1:**

: Abstract
Background:
First line: where was the utilization low? Incomplete sentence……. We have made a correction
Second line says no study while third line mention about the study: confuses the reader…..revised.

Method: need re-writing on the analysis part to have better clarity. For instance Bivariable and multivariable logistic regression analyses were also performed- for what? ... It is now revised.

Result: The result is confusing- particularly the interpretation of OR. …. It also modified for clarification.

Background

Lengthy explanations of technical issues, global issues- which could be shortened. …. We make now a revision on it.

Suggest adding up more context of Ethiopia. Even if the is now much study in Ethiopia we have made a revision now.

The statement of problem is kept separately which is not inline with the journal format. It is corrected
Methods

How many hospitals were selected? How were they selected? All hospital under the regional zone is included as source of population for study participants.

Sampling process is confusing. The author has calculated sample size as per objective assuming two outcomes (associations), which is fair but not sure how 354, was decided. In order to increase the number of samples we made a sampling calculation for the second objective (association) and we have got 354.

Was ethical approval obtained? Yes, from College of Medicine and health science IRB.

Result

The # of descriptive tables should be reduced and kept it as supporting tables. OK

Need to re-check the statistical data. For instance the interpretation says- Female health care providers who had good knowledge were nearly two times more likely to
utilize long-acting contraceptive methods than those who had poor knowledge [AOR=1.887, 95% CI: 1.020-3.491]- while its exactly opposite at the table. Some significant associations have very high CI intervals. For instance (CI: 5.065- 48.49) for ever-birth variable. It is corrected.

Discussion

Suggest comparing the data with the national scenario or other women groups in the country
Add up the strengths and expand the limitations
Add up recommendations to scale up the LACM uptake

We make a correction on these questions on strength, recommendation and weakness.

**Reviewer #2:**

1. The article bears a lot of resemblance to another published article with a similar topic but which took place in North West Ethiopia. We have tried to address new information on this study. This study justified to study on different area (new sites), background (religion, geography and multicenter working areas). It also added more variables from the previous study.
2. The presentation of the statistics should be improved, for example: proportion of the Health workers using LAFP is 33.6% was not shown in Figure 2, it is only available in Table 6. This should be clearly stated. Now it is clearly stated.
   It is not clearly shown how and the multi-variable logistic regression that was done in the analysis. This is stated clearly in **table 7**.
