# Supplementary material for: Utilization of long-acting contraceptive methods and associated factors among female healthcare providers in South Wollo Zone hospitals, Northeast, Ethiopia. A cross-sectional multicenter study
Source: PLOS Glob Public Health. 2023 Mar 27;3(3):e0001692. doi: 10.1371/journal.pgph.0001692 (PMC10042362; doi:10.1371/journal.pgph.0001692)
Supplement: Attachment — Submitted filename: A rebuttal letter of responses.docx [file pgph.0001692.s003.docx]

***A rebuttal letter of responses***:

Journal Requirements:

**Generally we have made language editing with native speaker. Some what our work is improved.**

1. Please review your reference list to ensure that it is complete and correct. If you have cited papers that have been retracted, please include the rationale for doing so in the manuscript text, or remove these references and replace them with relevant current references. Any changes to the reference list should be mentioned in the rebuttal letter that accompanies your revised manuscript. If you need to cite a retracted article, indicate the article’s retracted status in the References list and also include a citation and full reference for the retraction notice.

*We have no retracted reference list even though you have not indicated it on comments, but there is one thesis desertion reference which is highlight in revised manuscript.*

2. Please amend your detailed Financial Disclosure statement. This is published with the article. It must therefore be completed in full sentences and contain the exact wording you wish to be published.

a. State the initials, alongside each funding source, of each author to receive each grant.

b. If any authors received a salary from any of your funders, please state which authors and which funders.

If you did not receive any funding for this study, please simply state: *“The authors received no specific funding for this work.”*

*We made a correction for this as in the above said. “The authors received no specific funding for this work..*

3. Your current Financial Disclosure states, “Wollo University supported to conduct this study. The funders had no role in study design, data collection and analysis, decision to publish, or preparation of the manuscript”. However, your funding information on the submission form indicates that you did not receive funding. Please indicate by return email the full and correct funding information for your study and confirm the order in which funding contributions should appear. Please be sure to indicate whether the funders played any role in the study design, data collection and analysis, decision to publish, or preparation of the manuscript.

*Yes, this research is supported by Wollo University, but has no role in the study design, data collection and analysis, decision to publish, or preparation of the manuscript*.

4. In the online submission form, you indicated that "The data cannot be made publicly available for ethical or legal reasons. It will be available at request. The data will be available at request". All PLOS journals now require all data underlying the findings described in their manuscript to be freely available to other researchers, either 1. In a public repository, 2. Within the manuscript itself, or 3. Uploaded as supplementary information. This policy applies to all data except where public deposition would breach compliance with the protocol approved by your research ethics board. If your data cannot be made publicly available for ethical or legal reasons (e.g., public availability would compromise patient privacy), please explain your reasons by return email and your exemption request will be escalated to the editor for approval. Your exemption request will be handled independently and will not hold up the peer review process, but will need to be resolved should your manuscript be accepted for publication. One of the Editorial team will then be in touch if there are any issues.

*Now we are accepted the data availability within the manuscript itself, or uploaded as supplementary information.*

**Reviewers' comments’ response**

**Reviewer 1:**

Reviewer´s comments

Kindly edit the English. This paper needs to be edited by an English native speaker.

*Yes, we have tried editing this manuscript by a native language writer.*

“COR, CI, AOR”. Kindly write these in full and put abbreviations in brackets the first time you write about them. You can use abbreviations thereafter.

*COR- Crude odds ratio*

*AOR- Adjusted odds ratio*

*CI – Confidence Interval*

You used logistic regression analysis, but you don´t state anywhere whether you tested for multicollinearity. Did you check for multicollinearity?

*Yes! We have checked multicollinearity tests before we analyzed logistics regression.*

In table 3, you indicated that 43 out of 354 of the study participants have never “started sexual intercourse”, meaning they are virgins. You included the 43 virgins in your “LAPMS utilization practice analysis”. Why would a virgin use LAPM? LAPMs prevent pregnancy, why would a sexually inactive person use them?

*It does not mean that ‘43 out of 354 (n=311) of the study participants have never “started sexual intercourse”, rather their age at first sexual intercourse started (n=311) with the mean age 19.8 (SD ± 2.747) years. Already all are started sexual intercourse (n=354)*
