# Supplementary material for: Utilization of long-acting contraceptive methods and associated factors among female healthcare providers in South Wollo Zone hospitals, Northeast, Ethiopia. A cross-sectional multicenter study
Source: PLOS Glob Public Health. 2023 Mar 27;3(3):e0001692. doi: 10.1371/journal.pgph.0001692 (PMC10042362; doi:10.1371/journal.pgph.0001692)
Supplement: Attachment — Submitted filename: A rebuttal letter of responsess.docx [file pgph.0001692.s004.docx]

***A rebuttal letter of responses***:

Journal Requirements:

**Generally we have made language editing with native speaker. Some what our work is improved.**

1. Please review your reference list to ensure that it is complete and correct. If you have cited papers that have been retracted, please include the rationale for doing so in the manuscript text, or remove these references and replace them with relevant current references. Any changes to the reference list should be mentioned in the rebuttal letter that accompanies your revised manuscript. If you need to cite a retracted article, indicate the article’s retracted status in the References list and also include a citation and full reference for the retraction notice.

*We have no retracted reference list even though you have not indicated it on comments, but there is one thesis desertion reference which is highlight in revised manuscript.*

2. Reviewer #2: The major deficit of this manuscript is the English. I have consistently requested that the authors get a competent English copy editor versed in scientific writing to review the submission. It is very hard reading the texts with many errors in language. I noted that the major lapses are from page 10 to the end. The key information is there, but the use of English is poor. It has improved from the first submission, but not at a stage where this should be published.

*Now, we have made a lot of changes on language proofreading with a copy past editorial services with expert academic writers.*

3. Please ensure that the funders and grant numbers match between the Financial Disclosure field and the Funding Information tab in your submission form. Note that the funders must be provided in the same order in both places as well.

*“The authors received no specific funding for this work.”*

*We made a correction for this as in the above said. “The authors received no specific funding for this work.*

4. Your current Financial Disclosure states, “Wollo University supported to conduct this study. The funders had no role in study design, data collection and analysis, decision to publish, or preparation of the manuscript”. However, your funding information on the submission form indicates that you did not receive funding. Please indicate by return email the full and correct funding information for your study and confirm the order in which funding contributions should appear. Please be sure to indicate whether the funders played any role in the study design, data collection and analysis, decision to publish, or preparation of the manuscript.

*Yes, this research is supported by Wollo University, but has no role in the study design, data collection and analysis, decision to publish, or preparation of the manuscript*.
